# Customized Deep Learning Classifier for Detection of Acute Lymphoblastic Leukemia Using Blood Smear Images

**DOI:** 10.3390/healthcare10101812

**Published:** 2022-09-20

**Authors:** Niranjana Sampathila, Krishnaraj Chadaga, Neelankit Goswami, Rajagopala P. Chadaga, Mayur Pandya, Srikanth Prabhu, Muralidhar G. Bairy, Swathi S. Katta, Devadas Bhat, Sudhakara P. Upadya

**Affiliations:** 1 Department of Biomedical Engineering, Manipal Institute of Technology, Manipal Academy of Higher Education, Manipal 576104, Karnataka, India; 2 Department of Computer Science and Engineering, Manipal Institute of Technology, Manipal Academy of Higher Education, Manipal 576104, Karnataka, India; 3 Department of Mechanical & Industrial Engineering, Manipal Institute of Technology, Manipal Academy of Higher Education, Manipal 576104, Karnataka, India; 4 Manipal Institute of Management, Manipal Academy of Higher Education, Manipal 576104, Karnataka, India; 5 Manipal School of Information Science, Manipal Academy of Higher Education, Manipal 576104, Karnataka, India

**Keywords:** acute lymphoblastic leukemia (ALL), blood smear, convolutional neural networks, deep learning, white blood cells

## Abstract

Acute lymphoblastic leukemia (ALL) is a rare type of blood cancer caused due to the overproduction of lymphocytes by the bone marrow in the human body. It is one of the common types of cancer in children, which has a fair chance of being cured. However, this may even occur in adults, and the chances of a cure are slim if diagnosed at a later stage. To aid in the early detection of this deadly disease, an intelligent method to screen the white blood cells is proposed in this study. The proposed intelligent deep learning algorithm uses the microscopic images of blood smears as the input data. This algorithm is implemented with a convolutional neural network (CNN) to predict the leukemic cells from the healthy blood cells. The custom ALLNET model was trained and tested using the microscopic images available as open-source data. The model training was carried out on Google Collaboratory using the Nvidia Tesla P-100 GPU method. Maximum accuracy of 95.54%, specificity of 95.81%, sensitivity of 95.91%, F1-score of 95.43%, and precision of 96% were obtained by this accurate classifier. The proposed technique may be used during the pre-screening to detect the leukemia cells during complete blood count (CBC) and peripheral blood tests.

## 1. Introduction

ALL (acute lymphoblastic leukemia) is a lymphoid blood cell malignancy characterized by the development of immature lymphocytes [1]. These impaired white blood cells harm the entire body and bone marrow, putting the immune system as a whole at risk. It also inhibits the bone marrow’s capacity to generate red blood cells and platelets. Moreover, these cancerous cells can enter the bloodstream and cause serious harm to other regions of the human body, including the kidney, liver, brain, heart, and other organs, leading to the development of other deadly cancers. According to worldwide statistics by the World Health Organization (WHO)’s International Agency for Research on Cancer, they reported 437,033 cases of leukemia and 303,006 deaths as of 2022 [2]. The blood, bone marrow, and extramedullary sites all show signs of ALL. This deadly disease is categorized into T-lymphoblastic leukemia (Pre-T), B-lymphoblastic leukemia (Pre-B), and B lymphoblastic leukemia according to the WHO [3]. Mature-B lymphoblastic leukemia starts in the bone marrow and releases an abnormal quantity of white blood cells in the body. These dangerously formed cells are known as “leukemia cells” or “blasts” because they are severely undeveloped. Leukemia is defined by Khullar et al. [4] as an abnormal hyper-proliferation of polymorphonuclear leukocytes which do not produce solid tumor aggregates, i.e., liquid cancer. Acute leukemia cells mature fast and can be lethal if not detected early.

Machine learning plays a crucial part in the battle against various life-threatening diseases [5,6,7]. It also contributes significantly to educational and clinical studies [8,9,10,11]. Machine learning has shown significant potential in medical, engineering, psychology, multi-disciplinary science, earth sciences, analytical practices, healthcare, and other domains. Using blood smear images and CNN, this article provides an early filtering deep learning strategy for correctly identifying ALL. This algorithm can assist clinicians and medical personnel to a great extent. Acute lymphoblastic leukemia is a type of malignant blood cell cancer that affects mostly children and adults above age 65 [12]. Leukocytes, or white blood cells as they are commonly known, make up around one percent of all blood cells. It can be observed in the bone marrow, blood, and extramedullary sites. In this type of leukemia, the immature leukocytes proliferate in the body rapidly, bringing about the need for early detection. Usually, the diagnosis is done based on a bone marrow examination. This method is labor intensive, time-consuming, and might generate inaccurate results. Therefore, there is a need for automation. The peripheral blood smear (PBS) test is one of the methods used for screening for leukemia [13]. A blood samples smear is analyzed under the microscope. To automate the process of recognizing ALL, deep learning is already playing a significant role and will help in reducing manual error as well. Over the past, several strategies have been used to expedite the process of detecting leukemia using artificial intelligence. Automated analysis of PBS introduces a smart healthcare facility by screening a sample for diagnostic purposes [14,15].

The robustness and the performance of CNN inspired our research [16]. Therefore, we developed customized CNN architecture to classify ALL. According to the study, the accuracy and reliability of prediction have improved. In this research, we put forward a method to automate the process of lymphoblast (blast cell) detection in the single-celled image to help in the detection of leukemia. The C_NMC_2019 dataset was used to train the model, and evaluation was done on the same [17]. Deep learning eliminates the process of using hand-crafted features for classification. Instead, the classification is done using a CNN custom architecture. The average accuracy after 6-fold cross-validation on the reliable C_NMC_2019 dataset was 94.95%. A computer-aided diagnosis system can be built on this result, potentially reducing the time and error required for the same.

Over the years, several approaches have been utilized to automate the process of detecting ALL. Many of the methodological procedures resolve the utilization of various machine and deep learning algorithms. Jiang et al. [18] applied Vit-CNN ensemble models to diagnose ALL. An accuracy of 89% was obtained by the models, claims the study. Differentiate enhancement-random sampling (DEES) was used to prevent data imbalance. Leukemia subtypes identification using CNN and microscopic images were researched in [19]. Two image datasets, ASH-Image-Bank and ALL-IDB, were used. The accuracy obtained was 88.25% and 81.74%, respectively. This algorithm was compared with traditional ML algorithms, such as support vector machine, decision tree, and KNN. Ghaderzadeh et al. [20] used CNN to diagnose B-ALL and its further subtypes from peripheral smear images. Ten well-known CNN architectures were used. These models obtained good results with accuracy, specificity, and sensitivity of 99.85%, 99.89%, and 99.82%, respectively. The dataset was obtained from a Kaggle competition. Qiao et al. [21] used a compact CNN model for the preliminary screening of ALL. Two datasets, APL-Cytomorphology-JHH and APL-Cytomorphology-LMU, were used. They yielded a precision of 96.53% and 99.20%, respectively. Promyelocytes were distinguished from normal leukocytes in this study. A hybrid model that used mutual information was used to diagnose ALL [22]. The Deep CNN classifier achieved an accuracy of 98%. The AA-IDB2 database was considered for this study. The rest of the research is described in Table 1.

To further support our claim, the remainder of the article has been described in the following manner. The following section describes the workflow and image processing techniques used, and the network is explained. Section 3 presents the results produced by our classifier along with a comparison of the outcomes from other established studies. The conclusion of the article is described in Section 4.

## 2. Materials and Methods

**Figure 1:**
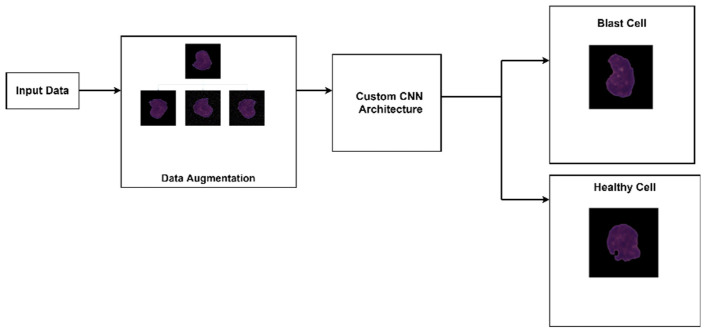
Leukemia screening system.

This section explains the workflow, image processing techniques, and network architecture developed for leukemia classification. Figure 1 describes the workflow followed for this work. After acquiring the dataset, augmentation was carried out to increase the size as well as the robustness. Afterward, the image data was fed to the CNN for automated feature extraction. The network then managed to classify the images as either ALL (blast cell) or HEM (healthy cell).

### 2.1. Input Data

**Figure 2:**
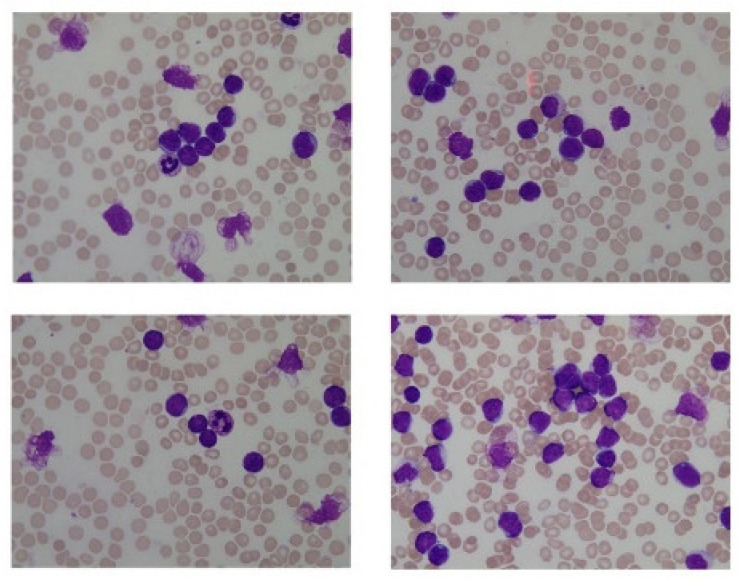
Original images.

**Figure 3:**
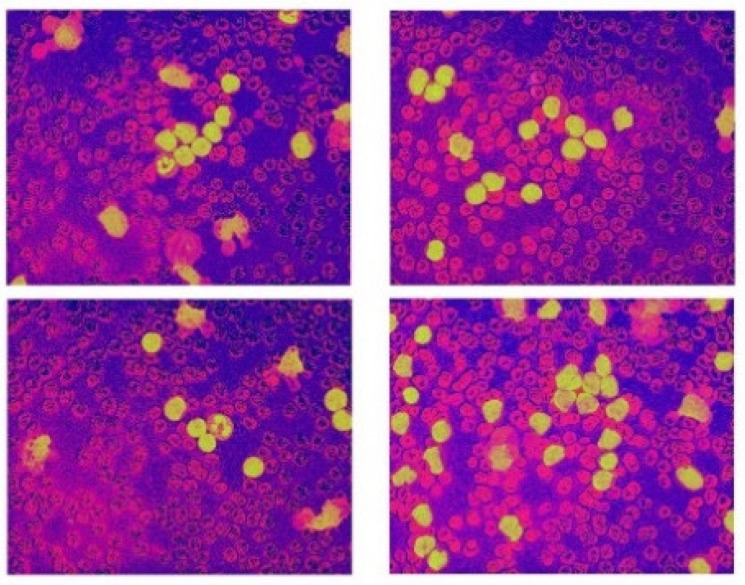
HIS color space.

**Figure 4:**
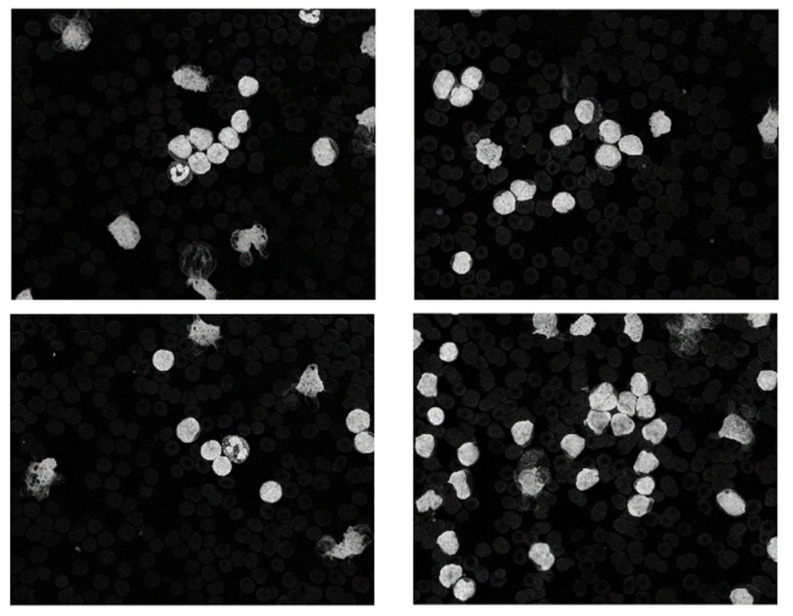
Saturation component.

**Figure 5:**
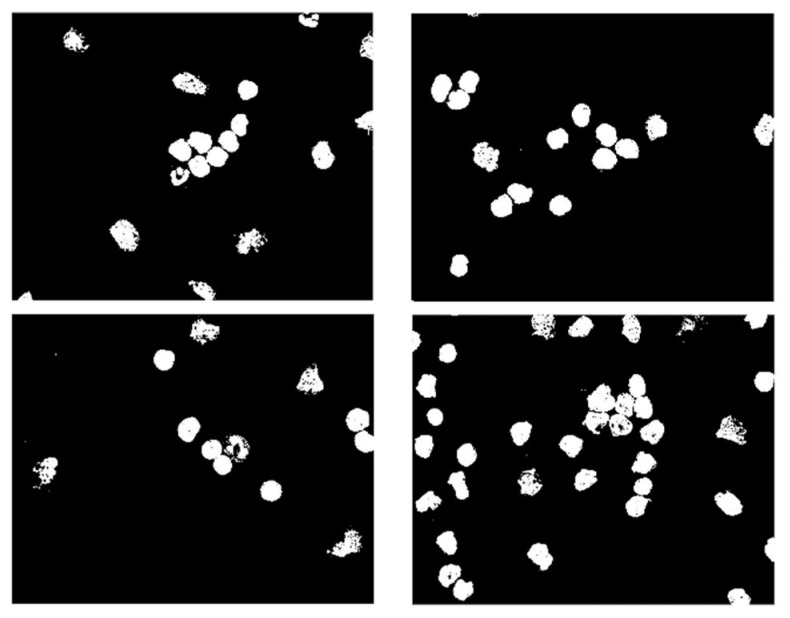
Images after thresholding.

**Figure 6:**
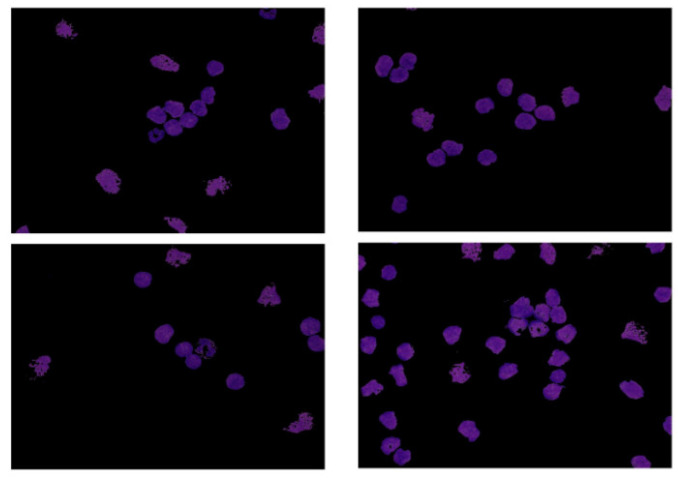
Image after segmentation.

The dataset used in this research belongs to the “ALL Challenge dataset of ISBI, 2019” [30,31,32,33]. The dataset contained cell images of both normal individuals and patients diagnosed with ALL. As shown in Figure 2, the original images acquired from a digital microscope having various components of the blood smear are pictorially depicted. The CNN classification for leukemia is done using segmented white blood cell (WBC) regions. This requires preprocessing and color segmentation. The subsequent process involves finding better segmentation of the region of interest. The HSI color space-based images of blood smears are shown in Figure 3. The white blood cells are seen to have better contrast than the other components of the image. To further localize the white blood cells, we selected the saturated component as it describes the intensity of the color, which is shown in Figure 4. This was then converted to a binary image by performing thresholding, as shown in Figure 5. The thresholding was performed on the gray scale image in such a way that all pixels in the range of (180–255) were converted to white, while all pixels belonging to values below this threshold were converted to black. The segmented image was obtained by finding the product of the original image and the segmented image, which was then used for further processing, as shown in Figure 6.

**Figure 7:**
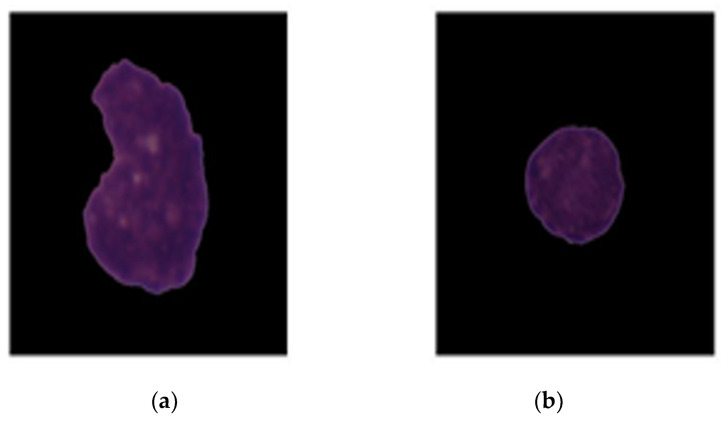
Images in the dataset: (**a**) represent blast cells; (**b**) represent healthy cell.

A total of 10,661 images were collected from 73 participants from the C NMC 2019 dataset. There were 7272 images of blast cells and 3389 images of healthy cells in total. The images in this dataset were uniform with a size of 450 × 450 × 3 and had been pre-processed such that only the object of interest (WBC) was included, and everything else was padded with black. Figure 2 gives a glimpse of the kind of image available in the dataset. Figure 7a represents the deadly blast cell, and Figure 7b represents a normal white blood cell. This dataset is reliable since expert oncologists have done the blast/healthy cell classification.

### 2.2. Data Augmentation

**Figure 8:**
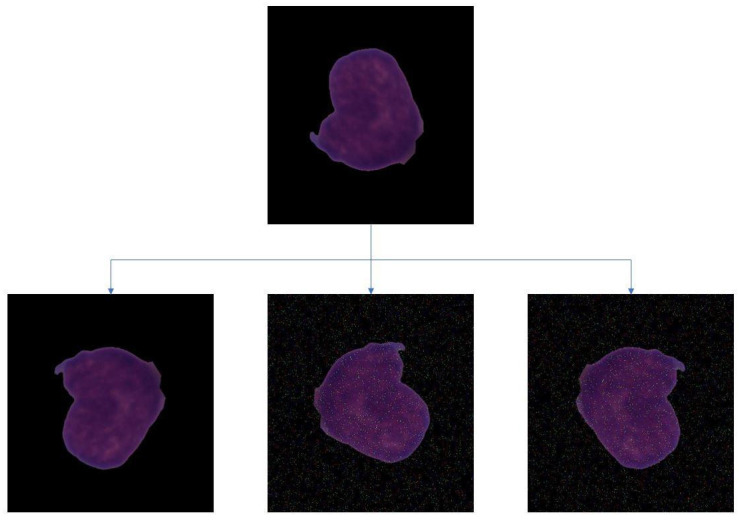
Original image of a blast cell and its augmented versions for the C_NMC_2019 dataset.

The number of images provided to the neural network plays a pivotal role in the feature extraction procedure. The dataset used had an imbalance of images of the two classes. This would make the classification process biased. So, to remove the bias, the images were subjected to auto orientation and resizing. Augmentation steps involved were: (1) vertical horizontal flipping, (2) clockwise and anti-clockwise rotation, (3) random brightness adjustments (4) random Gaussian blur with the addition of pepper and salt noise to the pixels. The final dataset consisted of 12,000 images, with 6000 images in each class. Figure 8 depicts the images obtained after augmentation.

### 2.3. CNN

A CNN has a sequence of layers that transforms an image volume into an output volume through a differential function. The architecture of CNN was inspired by the visual cortex of the brain. The architecture fits the data better because of the reduction in the number of parameters involved and the reusability of weights. There are different types of layers in a convolutional network which includes: convolution (CONV), pooling (Pool), and fully connected (FC).

- **Convolution (CONV) Layers**
  The convolution layers are the main building blocks of CNN. They comprise a set of independent filters, which are convolved with the input volume to compute an activation map made of neurons. The useful features from the input images are extracted by having multilayered architecture. Each of the filters can be of a different type, and they extract different features, such as vertical lines, horizontal lines, and edges. The CNN layers help in extracting features through convolution. The extracted deep features play a major role in the decision support system.

The 2D convolution is given by Equations (1) and (2), respectively.

(1) $$y\left[m,\;n\right]=x\left[m,\;n\right]\times h\left[m,\;n\right]$$

(2) $$y\left[m,\;n\right]=\sum_{i=-\infty }^{\infty }\sum_{j=-\infty }^{\infty }x\left[i,\;j\right].\;h\left[m-i,\;n-j\right]$$

where, *x*[*m*, *n*] = Input

*m*, *n* = no. of rows, no. of columns, respectively
*i*, *j* = row index and column index

Similarly, the size of the image after convolution is given by Equation (3):

(3) $$Size=\lfloor \left(\frac{m+2p-n}{s}+1,\;\frac{m+2p-n}{s}+1\right)\rfloor$$

where *m* = number of input features

*n* = convolution kernel size
*p* = padding
*s* = stride

**Figure 9:**
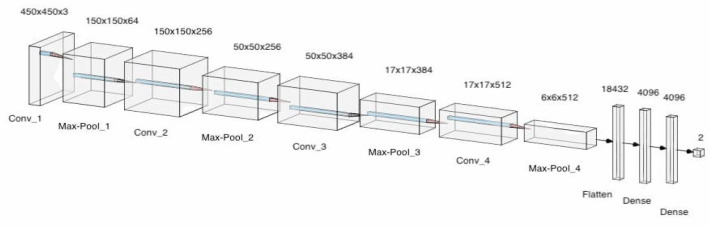
The architecture of CNN (ALLNet).

- **Pooling (POOL) Layer**
  Convolutional neural networks often use pooling layers to decrease the representation size and increase the speed of computation. A pooling layer summarizes the activities of local patches of nodes in the convolutional layers. Pooling can be done in two ways: max pooling and average pooling. In max pooling, the maximum value for each patch of the feature map is stored, while others are discarded. The intuition behind using max pooling is that the maximum value indicates that it has the most impact on that patch of the image. Hence other patches can be discarded. Average pooling follows a similar procedure, except in the place of the maximum value, the average value of the patch is taken, and all other values are discarded.
- **Fully Connected (FC) Layer**
  The FC input layer takes the output of the previous layers and turns them into a single vector that can be connected to the input layer of the next stage. This layer contains a softmax layer at the end, which predicts the correct label (0, 1). The output layer gives the final probability for each layer. The fully connected part of the CNN determines the most accurate weights by going through its backpropagation. The weights that each node receives are used to determine their respective labels. Since this project is of binary classification, the nodes will be prioritized to either 1 or 0.
- **Batch Normalization**
  Batch normalization decreases the covariance shift, i.e., the amount by which the hidden unit values shift. If the algorithm is trained to map some input *x* to some output *y*, and if the distribution of *x* changes, the prediction will not work as well, and retraining might be required. Batch normalization allows the learning of each layer in an independent manner. An advantage of using batch normalization is that learning rates can be set higher as it makes sure that no activation goes high or low. Batch normalization also reduces overfitting as it has regularization effects.
  To improve the stability of the neural network, batch normalization normalizes the previous activation layers’ outputs. This process adds two parameters to each layer, so the normalized output gets multiplied by gamma (standard deviation) and beta (mean).
  Mathematically, the mini-batch mean is given by Equation (4):
  (4) $$\mu_{B}=\frac{1}{m}\sum_{i=1}^{m}x_{i}$$
  Mini-batch variance is shown in Equation (5):
  (5) $$\sigma_{B}^{2}=\frac{1}{m}\;\sum_{i=1}^{m}\left(x_{i}-\mu_{B}\right)$$
  Normalization is given by Equation (6):
  (6) $$\hat{x_{i}}=\frac{x_{i}-\mu_{B}}{\sqrt{\sigma_{B}^{2}+\epsilon }}$$
- **Dropout**
  Deep neural networks are likely to overfit early on any given dataset. Dropout is a method of regularization that approximates training a large number of neural networks with different architectures in parallel. While training, some dropout layers are randomly ignored. This simulates the effect of a new layer, making the neural network treat it in such a way. In effect, each update is performed with a new outlook on the layer. This method makes the network more robust as additional noise is introduced.
- **Loss function**
  In the model, the categorical cross-entropy loss function has been implemented. The performance of this binary classification model, i.e., whose output lies between 0 and 1, is measured by the mentioned loss function. Categorical cross-entropy compares the distribution of the predictions with the true distribution, where the probability of the class in consideration is set to 1 and the probability of the other classes is set to 0.
  Categorical cross-entropy is shown in Equation (7):
  (7) $$L\left(y,\;\hat{y}\right)=-\sum_{j=0}^{M}\sum_{i=0}^{N}\left[y_{ij}\times \log\left(\hat{y_{ij}}\right)\right]$$
  where *y*-hat is the predicted expected value and y is the observed value.
- **Optimizer**
  Optimizers are algorithms used to change the attributes of the neural network such as learning rates and weights to reduce the loss. In the model, adaptive movement estimation (Adam) has been incorporated. Adam is a combination of the root mean square propagation (RMSProp) and adaptive gradient algorithm (AdaGrad). The proposed convolution neural network architecture is shown in Figure 2, which makes use of pooling layers, fully connected layers, convolutional layers, dropout and batch normalization. Features were automatically extracted from the input images by the CNN. Feature extraction is then performed by the convolutional layers and the pooling layers. Four convolutional layers, four max-pooling layers, and 3 fully connected layers were utilized. Batch normalization and Dropout were applied to account for overfitting, vanishing, and exploding of gradients. This model consisted of 95,099,266 parameters in total. The architecture for the designed model can be seen in Figure 9. A more detailed description of the model is described in Table 2.

## 3. Results and Discussion

### 3.1. Performance Metrics

The performance metrics estimated include *accuracy*, *precision*, *recall*, and *F*1 *score*:

***Accuracy***: It is the ratio of true positive predictions to the total number of predictions and is given in Equation (8).

(8) $$Accuracy=\frac{True\;positive+True\;Negative}{Total\;samples}\times 100$$

***Precision***: It is the ability of the model to return only relevant instances, given by Equation (9).

(9) $$Precision=\frac{True\;Positive\;\left(TP\right)}{True\;Positive\left(TP\right)+False\;Positive\;\left(FP\right)}\times 100$$

***Recall***: It is the ability of the model to identify all relevant instances, as shown in Equation (10). It emphasizes false negative results. This is also called the *true positive* rate or sensitivity.

(10) $$Recall=\frac{True\;positive\;\left(TP\right)}{True\;positive\;\left(TP\right)+FalseNegative\;\left(FN\right)}\times 100\;$$

***Specificity*:** It is an important metric to identify false-positive results. It is described in Equation (11)

(11) $$Specificity=\frac{True\;negative\;\left(TN\right)}{True\;negative\;\left(TN\right)+FalsePositive\;\left(FP\right)}\times 100$$

***F*1 *Score***: This is the harmonic mean of precision and recall and is used to indicate a balance between *Precision* and *Recall* given in Equation (12).

(12) $$F1\;Score=2\times \frac{Precision\times Recall}{Precision+Recall}$$

### 3.2. Model Evaluation

The ALL-NET architecture consists of a total of 4 convolution layers alternated with max pooling layers. This setting is followed by a total of 3 fully connected layers. The reason for keeping a max pooling layer after every convolution is to maintain the size of the processed image instance to a minimum. This can create a potential problem; if excessive max pooling is done, then it can lead to potential loss of information or patterns which are not spanning wide or large enough. Further, as observed previously, the data augmentation step has already added noise to the image; such noise can also affect the operation of max pooling. Batch normalization is carried out during every alternate max pooling to make sure that the flowing data is normalized, and every neuron has some input to give. Finally, to avoid any overfitting, we use dropout. These layers will ensure that multiple neurons having similar weight vectors are not unintentionally learning the same pattern in the given image instance. The learning rate was initially set at 1 × 10^−3^ initially, but it was observed that 1 × 10^−5^ gave marginally better improvement during the learning phase. The batch size for the training images was set at 16. While the epochs were initially selected to be in the range of 50 to 100. As pointed out in the later sections, it was found that the model was prone to overfitting if the epochs exceeded 70. Further fine tuning was done, and 65 epochs were finally selected as the final parameter value.

**Figure 10:**
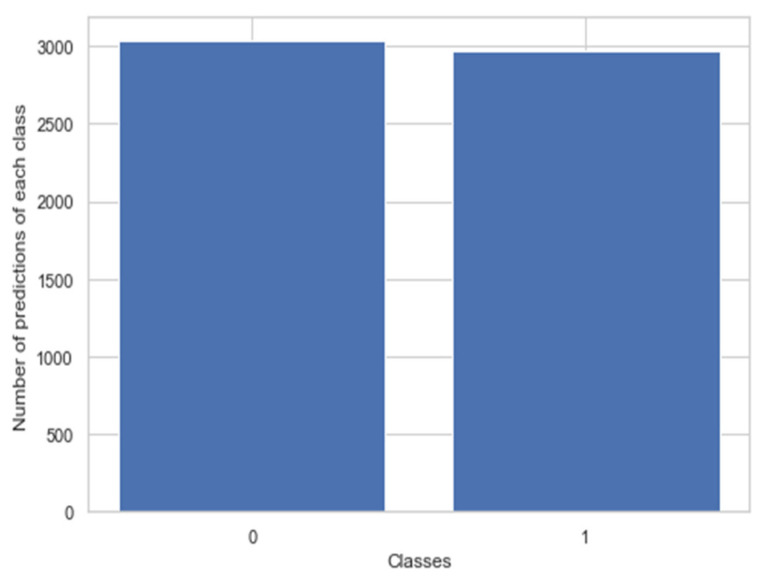
Prediction of classes of the test set images.

After image augmentation, the number of test instances available as blast cell images and healthy cell images was nearly the same as shown in Figure 10, so there was no imbalance present.

**Figure 11:**
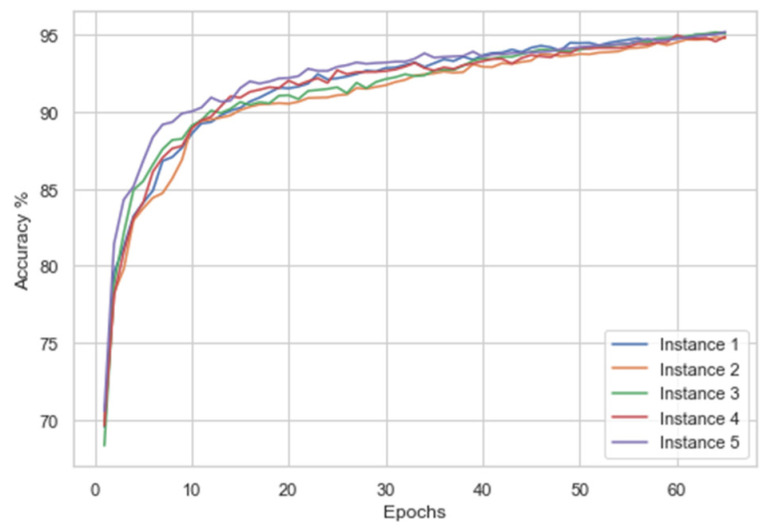
Accuracy for the five instances.

**Figure 12:**
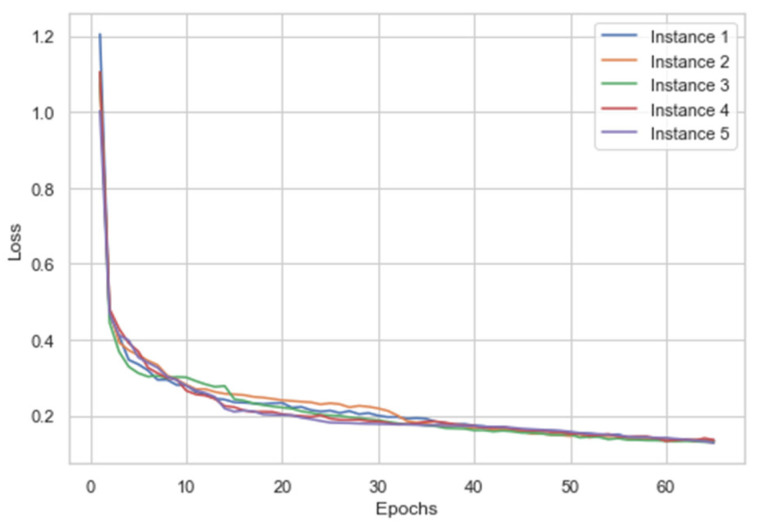
Training loss for the five instances.

The dataset was initially given an 80–20 train test split. The training split was further divided into 5-fold cross-validation. The best performing fold was then utilized for testing on the holdout validation 20% test split. The cross-validation was carried out to overcome any potential overfitting which might occur due to model exposure to one class of images. The proposed model was trained on 12,000 images and tested on 2132 images. Each fold of 5-fold cv was learned by the model for 65 epochs. We performed validation of this model by training and conducting simultaneous test evaluations for five runs. The augmented data was mixed along with the original data to provide a wide range of training samples, this in turn helped the model to generalize better. The accuracy and loss curves are described in Figure 11 and Figure 12, respectively. From Figure 12, it can be noticed how the categorical cross-entropy loss function starts to reduce heavily in the span of 10–30 epochs, referring to Figure 11. The model is performing adequately but stopping the model training at this point would have led to potential underfitting. Epochs after 35 do not provide many variations in the loss, and a steady decrease in the loss function can be observed. This behavior of the ALL-NET was observed in all 5 separate training processes. While increasing the epoch number from 65 would have led to a decrease in the loss even further as well as simultaneous increase in the accuracy, this would have led to overfitting as we will see when the model is evaluated against the holdout test set.

During the first instance, the accuracy, specificity, recall, F1-score, and specificity were 94.94%, 94.87%, 94%, 94.96%, and 95.95%, respectively. When the model was run again, the accuracy, specificity, recall, F1-score, and specificity obtained were 94.5%, 95.8%, 93.2%, 93.2% and 96%, respectively. When the model was run the third time, the above metrics obtained were 94.72%, 95.8%, 93.2%, 93.2%, and 96%, respectively. During the fourth iteration, the accuracy, specificity, recall, F1-score, and specificity obtained were 95%, 95.91%, 94%, 94.96 and 95.95%, respectively. In the last instance, the above metrics obtained were 95.45%, 95%, 95.91%, 95.43% and 94.94%, respectively. In Figure 12, the accuracies are plotted against epochs.

**Figure 13:**
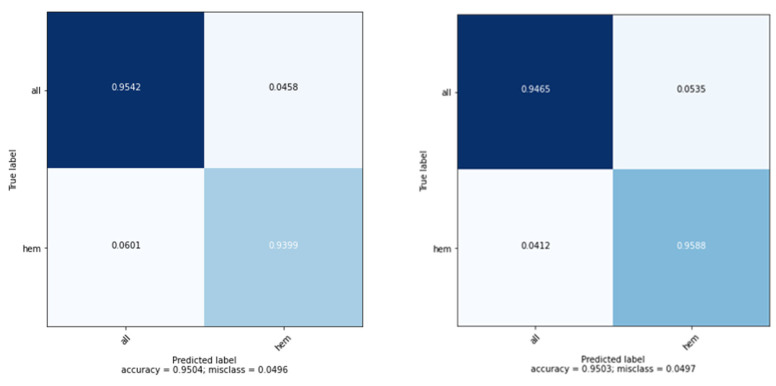

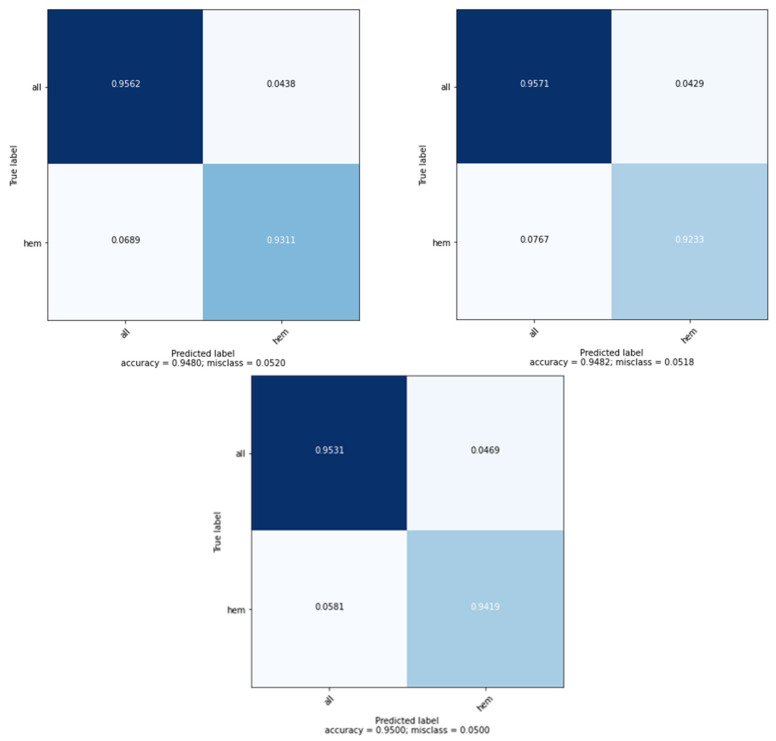
Confusion matrix of 5 separate training instances.

A confusion matrix was used to evaluate the number of true predictions. The prediction was done on two possible classes, “ALL”, i.e., blast cells and “HEM” i.e., healthy cells. It was found that from a total of 2132 images, 1454 images were classified as blast cells, and 667 images were classified as healthy cells. During prediction, the model predicted 1996 images, i.e., 94.2% images correctly, and 5.8% images incorrectly. Predictions made per class are seen more clearly in Figure 13, depicting the confusion matrix. These results can be seen in Table 3.

Referring to Table 3, it can be observed that the F1 score obtained by the model in each of the 5 running instances is up to par if not better than the current existing model performances which we shall discuss later. A consistent F1 score of 90 plus in all 5 runs indicates that the balance between the precision and recall is maintained. The balance between precision and recall is quite pertinent for a model such as ALL-NET which can potentially serve as a preliminary screening tool. Our approach of using a simple CNN network for this dataset has given us good results across multiple performance metrics.

Many approaches have been used for classifying leukemia. Of the many, a few approaches with good performance are discussed in Table 4. ALL was diagnosed using Abunadi et al. [34] using an ensemble deep learning approach. The combined model achieved an accuracy of 100%. For the C_NMC_2019 dataset, Yongsheng Pan et al. [35] used a neighborhood correction technique to diagnose this fatal condition at an early stage. An accuracy of 92% was obtained by this algorithm. Khandekar et al. [36] used the YOLOe4 algorithm to diagnose this fatal disease. A maximum recall of 96% was obtained by the deep learning model. Christian et al. [37] have utilized an attention-based neural network to detect ALL. A maximum F1-score of 82% was obtained by this efficient algorithm. Table 4 demonstrates the comparison of prior research with the suggested technique for a similar dataset. The need for preprocessing to tackle this problem may be a slight drawback in this research. The model could be made more robust if it were trained with more data. The intelligent algorithms are promising with the optimized features for screening various problems in digital pathology [38,39,40]. The improved telehealth framework with the intelligent algorithm will enable the remote diagnosis facility [41,42,43].

## 4. Conclusions

A method for early diagnosis of cancer from infinitesimal images of white blood cells using CNN has been proposed in this research. Since the deep learning approaches do not require manual feature engineering, the model performs exceptionally well when compared to traditional image processing techniques. The good performance of blast cell detection is supported by the accurate classification results. Maximum accuracy of 95% was obtained by the custom deep learning ALL-NET classifier. It operates on all data available rather than a portion specified by a feature vector, which is also a benefit. This work can help during the screening, reducing the rate of error, as well as decreasing the computational time. As a result, this research can be used to provide a theoretical framework for a diagnosis support tool for the detection of ALL. The future study includes expanding the dataset with noisy images with very little pre-processing, to address the problem of using actual medical images for prediction. Combining these models with explainabality models provides useful inferences to practitioners. Algorithms such as Yolov4, Resnet, and AlexNet can also be explored since they can perform better on these tasks.

## Figures and Tables

**Table 1:** Existing research that diagnoses ALL using deep learning.

Research	Dataset	Algorithm Used	Accuracy	Precision	Recall
[23]	Three hybrid image databases	CNN and SVM	99%	99%	99%
[24]	IDB dataset	AlexNet	96%	-	96.74%
[25]	DNA Sequence images	CNN and other ML models	75%	-	-
[26]	GRTD dataset	VCGNet	96%	93%	93%
[27]	BCCD ALL-IDB2 JTSC CellaVision databases	CNN	97%	80%	94%
[28]	LISC and Dhruv dataset	CNN	97%	80%	94%
[29]	Amreek Clinical Laboratory	CNN	97.75%	-	-

**Table 2:** ALLNet Architecture.

Layer (Type)	Layer Shape	Number of Parameters
Conv2D	(450, 450, 3)	1792
Max_Pooling_2D	(150, 150, 64)	-
Conv2D	(150, 150, 256)	147,712
Max_Pooling_2D	(50, 50, 256)	-
Conv2D	(50, 50, 384)	885,120
Batch_Normalization	(50, 50, 384)	1536
Max_Pooling	(17, 17, 384)	-
Dropout	(17, 17, 384)	-
Conv2D	(17, 17, 512)	1,769,984
Batch Normalization	(17, 17, 512)	2048
Max_Pooling	(6, 6, 512)	-
Dropout	(6, 6, 512)	-
Flatten	18,232	-
Dense	4096	75,501,568
Dropout	4096	-
Dense	4096	16,781,312
Dropout	4096	-
Output	2	8194

Total Parameters:—95,099,266, Trainable Parameters:—95,097,474, Non-Trainable Parameters:—1792.

**Table 3:** Model evaluation results.

Instance	Accuracy (%)	Specificity (%)	Recall (%)	F1-Score (%)	Precision (%)	Matthews Correlation Coefficient (MCC)
Instance 1	94.94	94.87	94	94.96	95.95	89.42
Instance 2	94.5	95.8	93.2	93.2	96	89.53
Instance 3	94.72	95.8	93.2	93.2	96	88.76
Instance 4	95	95.91	94	94.96	95.95	88
Instance 5	95.45	95	95.91	95.43	94.94	89.5

**Table 4:** Comparison of results on the C_NMC_2019 dataset.

Reference	Method	Salient Features	Performance Measure
[34]	Bagging Ensemble with Deep Learning	Bagging ensembling was utilized	F1-score of 88%
[35]	Neighborhood Correction Algorithm	Fine-tuning a pre-trained residual network, constructing a Fisher vector based on feature maps, and correction using weighted majority.	F1-score of 92%
[37]	CNN	Regional proposal subnetwork.	F1-score of 83%
Proposed	CNN	Improved accuracy	F1-score of 96%

## Data Availability

Not applicable.
